# The current status and influencing factors of core competencies among China’s internet-based “sharing nurses” based on social-ecological system theory: a multicenter cross-sectional study

**DOI:** 10.3389/fmed.2025.1693463

**Published:** 2025-12-09

**Authors:** Wang Yudong, Xing Zhaowei, Jiao Linlin, Zhang Yishu, Wu Jianghua, Yang Linlin, Yang Chunling

**Affiliations:** 1 Department of Nursing, Liaocheng People’s Hospital, Medical School of Liaocheng University, Liao’cheng, Shandong Province, China; 2 School of Nursing, Shandong First Medical University, Tai’an, Shandong Province, China; 3 Department of Nursing, Liaocheng People’s Hospital, Liao’cheng, Shandong Province, China; 4 School of Nursing, Soochow University, Su’zhou, Jiangsu Province, China

**Keywords:** sharing nurses, the social ecosystems theory, core competencies, emergency response competencies, nurses

## Abstract

**Aims:**

This study aims to investigate the current status of core competencies and emergency response competencies among Chinese sharing nurses, examine the correlation between core competencies and emergency response competencies, and identify the factors influencing the core competencies of these nurses.

**Methods:**

This multicenter cross-sectional study was conducted in July 2025 at 6 hospitals in 6 cities in Shandong Province, with 1,011 nurses participating in the study. Data were collected using the following tools: (1) a socio-demographic questionnaire, (2) the Core Competency Assessment Tool for “sharing nurses,” and (3) evaluation index system for emergency response competencies of “Internet+” nurses. SPSS 26.0 was used to analyze the data, *Spearman* correlation analysis was used to evaluate the correlation between the core competencies of “sharing nurses” and emergency response competencies, and linear regression was used to explore the factors influencing the core competencies of these nurses.

**Results:**

Among the 1,011 participating nurses, 41.05% had delivered more than one Internet-Based nursing service. A significant positive correlation was observed between the core competencies of “sharing nurses” and their emergency response competency scores (*r* = 0.852; *p* < 0.001). The level of “sharing nurses” core competencies was influenced by the following factors: hospital level, hospital leadership support, team therapeutic support, family support, nurse personality, subjective willingness, training experience, and emergency response competencies.

**Conclusion:**

Chinese “sharing nurses” demonstrated a high level of core competencies and emergency response abilities, and the two are closely related. Based on these findings, healthcare institutions should actively provide standardized training in accordance with national policies, with an emphasis on enhancing nurses’ subjective willingness to become “sharing nurses” and promoting the actual delivery of nursing services. Supported by hospital-based therapeutic teams, these efforts will help safeguard the quality of life and safety of home-based patients, while also providing a foundation for refining the “Internet+ nursing service” model and optimizing the allocation of primary healthcare human resources.

## 1 Introduction

The nurses’ core competencies serve as the “gold standard” for evaluating nursing practice proficiency, reflecting the fundamental knowledge and skills expected of all nurses regardless of their specialization (1). Globally, enhancing nurses’ core competencies has become a critical objective in health system development. Particularly in middle-and low-income countries, critical thinking, research capability, clinical care, and professional development are not only regarded as essential core competencies but also represent more pressing educational needs (2).

The proportion of China’s older adult population is increasing every year. Consequently, for families providing home care for the older adult, the demand for specialized medical care, rehabilitation, and home care services will increase substantially (3). Furthermore, regional development disparities have resulted in a relative shortage of registered nurses in rural China, along with a scarcity of highly educated and high-ranking personnel, the quantity and quality of nurses are insufficient to effectively meet societal demands (4). Amid rising healthcare needs and increasing patient demand for personalized nursing services, and to proactively address population aging, implement the Healthy China strategy, and expand nursing service provision, China’s National Health Commission has further advanced the pilot program of “Internet+nursing service,” requiring health commissions at all levels to coordinate regional medical resources to enhance primary-level nursing service capacity (5). The National Nursing Care Industry Development Plan (2021–2025), issued by China’s National Health and Health Commission (6), explicitly encourages medical institutions to innovate their nursing care service models based on hierarchical diagnosis and treatment and the actual needs of the population, promoting the active provision of “sharing nurses” services, whereby registered nurses deliver home-visit nursing services to patients through Internet platforms. This model, in which registered nurses provide home-based care through Internet platforms, extends professional nursing services to households, communities, and rural areas. It serves discharged patients, the older adult, and other mobility-impaired populations, demonstrating significant potential to meet personalized care needs, alleviate pressure on the healthcare system, promote hierarchical diagnosis and treatment, and facilitate the dissemination of high-quality medical resources (7). However, “Sharing Nurse” services are predominantly delivered independently in home or community settings, which may lead to insufficient communication with patients’ primary medical teams. Furthermore, the absence of standardized training protocols across different medical institutions means some “sharing nurses” may lack experience or specialized skills, leading to heterogeneous service quality (8). Concurrently, health regulatory authorities possess insufficient understanding of the current status and influencing factors of “sharing nurses” core competencies and emergency response competencies. This knowledge gap hinders the development of effective regulatory standards, constituting a significant factor affecting the quality of Internet-based nursing services (9).

Therefore, this study employs a cross-sectional design to investigate the current status of core competencies among Chinese “sharing nurses” and analyze their influencing factors, while exploring the potential correlation between these core competencies and emergency response capabilities. The findings are expected to offer an empirical foundation for health authorities, nursing managers, and educators to formulate targeted training strategies, improve the professional capacity of “sharing nurses,” enhance their sense of occupational achievement and motivation for growth, expand the competent workforce in this field, and ultimately contribute to high-quality development in the nursing industry.

## 2 Theoretical framework

**Figure 1:**
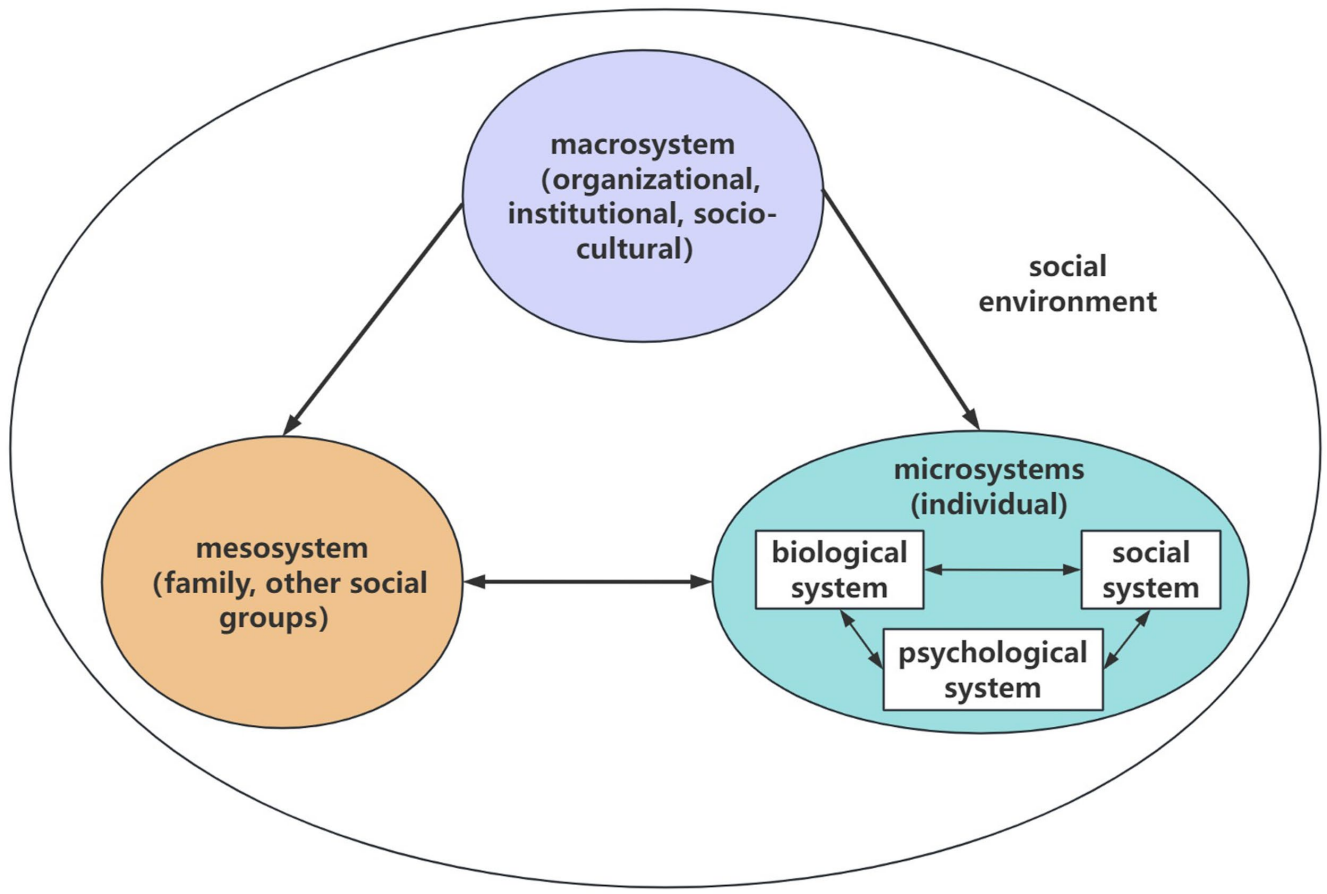
The social ecosystems theory.

The Social Ecosystems Theory (SET) (10) has gained increasing prominence in the field of health management, this framework facilitates understanding of the interactions among social, economic, environmental, and individual factors underlying health issues, emphasizing the reciprocal relationship between systems and individuals and its influence on individual development. SET categorizes human social ecosystems into three fundamental levels: microsystem, mesosystem, and macrosystem. The microsystem encompasses the individual and their immediate biological, psychological, and social subsystems that shape behavior. The mesosystem comprises small-scale social structures, such as families and peer groups. The macrosystem represents broader societal structures, including organizations, institutions, and socio-cultural contexts. Currently, SET is applied to analyze factors influencing chronic diseases, understand caregiver experiences, and guide health promotion efforts, serving as an important theoretical guide (Figure 1).

## 3 The study

### 3.1 Aim

The objectives of this study are: (1) to investigate the current status of core competencies among nurses providing “Internet-Based Nursing Services” in Shandong Province; (2) to explore the correlation between core competencies and emergency response competencies in this nurse population; (3) to analyze the factors influencing the core competencies of these nurses.

### 3.2 Study design

This study was a cross-sectional study and the study design followed the STROBE Statement (11) for cross-sectional studies.

### 3.3 Participants

This study was conducted in July 2025 at 6 hospitals in 6 cities in Shandong Province. The inclusion criteria for nurses were: (1) serving nurses; (2) age≥18 and ≤65 years; (3) voluntary participation in this study. The exclusion criteria was non-on-the-job personnel. The exclusion criteria was: (1) logical errors in the general information, for example: length of working hours - length of becoming a “sharing nurses” ≤ 4 years (the China Health and Wellness Administration Commission requires nurses to have 5 or more years of work experience for “sharing nurses” practice) (12).

### 3.4 Sample size

**Figure 2:**
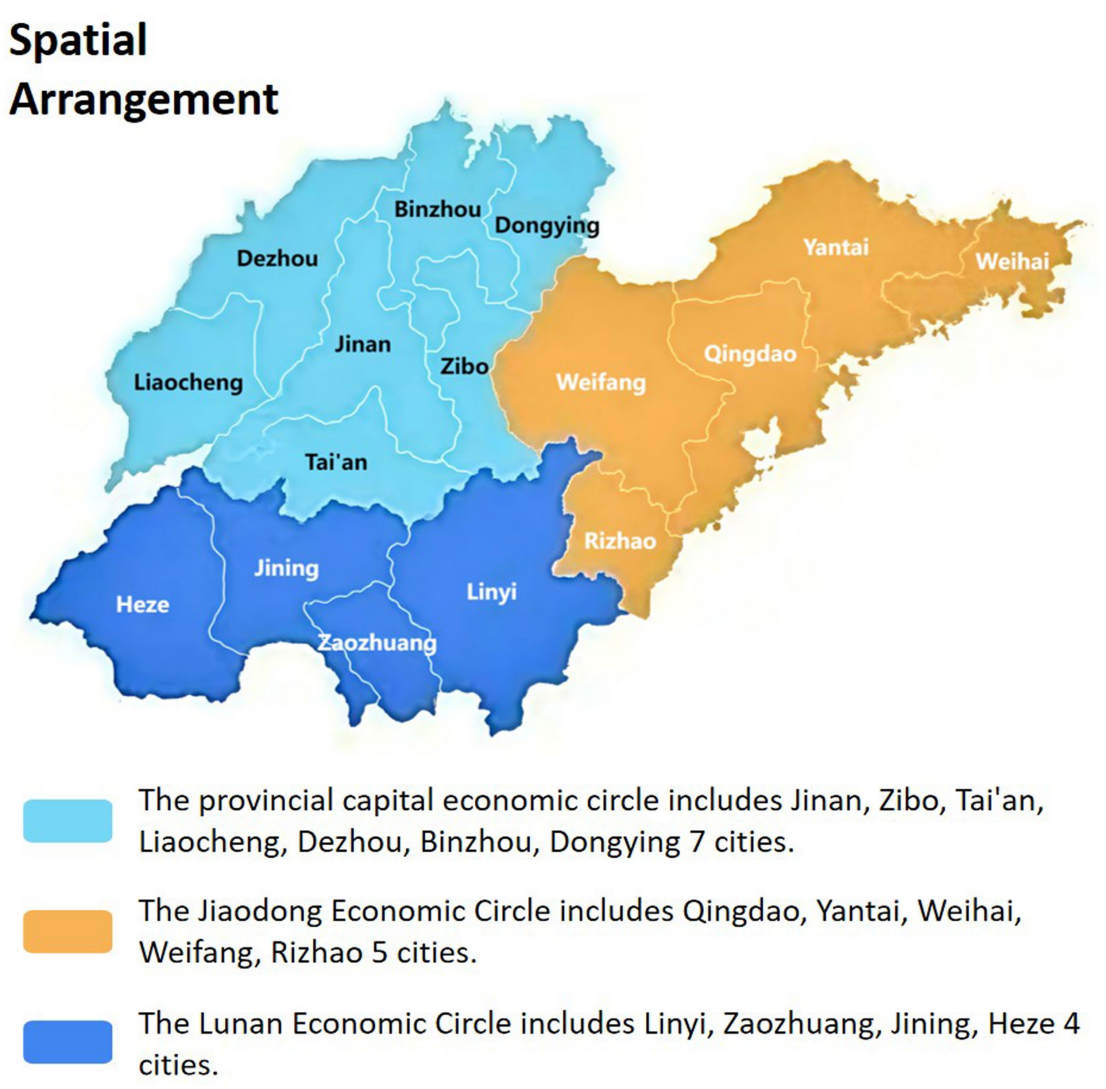
Shandong Province Economic Circle division map.

This study was conducted in Shandong Province, China. According to the 2020 Seventh National Population Census Bulletin (13) data, Shandong has a population exceeding 100 million, with individuals aged 60 years old and above accounting for 20.90%. A multi-stage stratified sampling method was employed. In the first stage, 2 cities were randomly selected from each of three geographic regions (Figure 2: Provincial Capital Economic Circle, Jiaodong Economic Circle, and Lunan Economic Circle). In the second stage, 1 hospital was randomly selected within each chosen city, yielding a total of 6 hospitals for the survey. Referring to the research results of Xiaofan (14), 282 nurses scored 227.20 ± 27.19 on the scale of core competencies evaluation index system of “sharing nurses.” According to the formula for estimating the mean of continuous variables $n=\frac{Z_{1-\alpha /2}^{2}\cdot \sigma^{2}}{E^{2}}\cdot \text{DEFF}$ , where: *Z*_1-*α*/2_ = *Z*-value for the 95% confidence level (*α* = 0.05, hence *Z* = 1.96); *σ* = Population standard deviation, set at 27.19 based on Xiaofan (14); *E* = Allowable error, set at 5 (typically < *σ*/5); DEFF = Design effect, conservatively set at 2. Accounting for an anticipated 20% invalid questionnaires, the calculated minimum effective sample size was 285 cases. In this study, 1,011 cases of valid questionnaires were actually returned, and the design effect and precision met the requirements.

### 3.5 Research tools

1. Socio-demographic information questionnaire: self-designed according to the purpose and content of the study by reading the literature combined with expert opinions. This study investigated the general information of the participants, including personality, education level, per capita household disposable income (According to the 2024 classification standards of China’s National Bureau of Statistics, China’s per capita disposable income for households stands at CNY 41314/USD 5763), etc. (Table 1).
2. The core competencies evaluation index system of “sharing nurses”: The evaluation index system for core competencies of network nurses developed by Xiaofan et al. (14), it contains six dimensions, including home-based nursing practice ability, critical thinking ability, communication and coordination ability, management ability, self-protection ability and professional development potential, and the higher the score. Higher scores indicate greater core competency. The scale was based on a 5-point Likert scale, with scores ranging from 1 (“Very inconsistent”) to 5 (“Very consistent”). Total scores range from 54 ~ 270 points, which can be grouped into lower level (54 ~ 162 points), moderate level (127 ~ 199 points), and high level (200 ~ 270 points). Its overall Cronbach’s *α* coefficient was 0.976, folded reliability was 0.895, and content validity index was 0.974.
3. Evaluation index system for emergency response competencies of “Internet+” nurses: developed by Huang et al. (15) based on the 4R crisis management theory framework, this scale assesses four dimensions of nurses’ emergency response competency: preventive competency, preparedness competency, response competency, and restorative competency. The higher the score indicates the higher level of “sharing nurses” emergency response ability. The scale comprises 40 items, using Likert 5-level scoring method, from “very poorly done” (1 point) to “very well done” (5 points). The total score ranges from 40 ~ 200 points, with higher scores representing a higher level of emergency response competency. The Cronbach’s alpha coefficient of the scale was 0.983, the half reliability was 0.916, and the content validity index was 0.970.

**Table 1:** Sample characteristics and univariate analysis.

Variable		Median (interquartile range)	*N* (%)	Scores for the core competencies evaluation index system of “sharing nurses”	Statistics	*P*
Hospital level	Second-class A		296 (29.28)	244.50 (216.00, 270.00)	52.885^a^	<0.001
	Third-class B		316 (31.26)	268.00 (248.00, 270.00)		
	Third-class A		399 (39.46)	257.00 (219.00, 270.00)		
Gender	Female		932 (92.19)	261.00 (218.25, 270.00)	−1.269^b^	0.204
	Male		79 (7.81)	268.00 (217.00, 270.00)		
Age		36.00 (34.00, 40.00)			0.132^c^	<0.001
Ethnicity	Han		1,001 (99.01)	261.00 (218.00, 270.00)	−0.707^b^	0.479
	Other		10 (0.99)	268.00 (230.75, 270.00)		
Educational level	Technical secondary school degree and below		1 (0.10)	Constant:255	3.936^a^	0.268
	Associate degree		30 (2.97)	255.50 (216.00, 270.00)		
	Bachelor degree		960 (94.95)	261.00 (218.00, 270.00)		
	Master degree and above		20 (1.98)	269.50 (241.00, 270.00)		
Whether an only child	No		881 (87.14)	261.00 (218.50, 270.00)	-0.424^b^	0.672
	Yes		130 (12.86)	264.00 (216.00, 270.00)		
Marital status	Unmarried		65 (6.42)	261.00 (218.50, 270.00)	−1.295^b^	0.195
	Married		946 (93.58)	264.00 (216.00, 270.00)		
Number of children	None		72 (7.12)	229.50 (213.50, 270.00)	0.059^c^	0.063
	1		316 (31.26)	258.50 (218.00, 270.00)		
	2		588 (58.16)	263.50 (221.00, 270.00)		
	3		34 (3.36)	266.00 (216.00, 270.00)		
	4		1 (0.10)	Constant:261		
Demographic characteristics	Rural areas		423 (41.84)	260.00 (216.00, 270.00)	−1.706^b^	0.088
	Urban areas		588 (58.16)	262.00 (224.00, 270.00)		
Household disposable income per capita	<41,314 CNY (5763USD)		426 (42.14)	261.00 (216.00, 270.00)	-1.010^b^	0.132
	≥41,314 CNY (5763USD)		585 (57.86)	262.00 (221.00, 270.00)		
Personality	Introverted		547 (54.10)	250.00 (216.00, 270.00)	−6.203^b^	<0.001
	Extroverted		464 (45.90)	267.00 (233.00, 270.00)		
Employment method	labor dispatch		4 (0.40)	269.50 (188.75, 270.00)	0.435^a^	0.933
	Personnel agency		348 (34.42)	261.00 (220.25, 270.00)		
	Contract		463 (45.80)	263.00 (216.00, 270.00)		
	Career-based/registered system		196 (19.38)	260.00 (221.25, 270.00)		
Title	Junior nurse		39 (3.86)	263.00 (215.00, 270.00)	11.405^a^	0.022
	Senior nurse		198 (19.58)	265.00 (216.75, 270.00)		
	Charge nurse		685 (67.75)	260.00 (217.00, 270.00)		
	Associate nurse practitioner		83 (8.21)	268.00 (245.00, 270.00)		
	Chief nurse practitioner		6 (0.60)	261.00 (238.75, 264.75)		
Position	No		877 (86.74)	261.00 (216.00, 270.00)	14.508^a^	0.006
	Deputy head nurse		65 (6.43)	253.00 (219.00, 270.00)		
	Head nurse		63 (6.23)	268.00 (255.00, 270.00)		
	Head nurse of major department		3 (0.30)	232.00 (162.00, 263.00)		
	Deputy director of nursing department		0			
	Director of nursing department		3 (0.30)	256.00 (241.00, 268.00)		
Duration of clinical experience (years)		13.00 (10.00, 17.00)			0.115^c^	<0.001
Work Department	Internal medicine		460 (45.50)	263.50 (220.00, 270.00)	14.470^a^	0.013
	Surgery medicine		258 (25.52)	263.50 (223.75, 270.00)		
	Gynecology and pediatrics		96 (9.50)	257.00 (216.00, 270.00)		
	ICU and emergency department		103 (10.19)	264.00 (216.00, 270.00)		
	Outpatient clinics, medical technology, and auxiliary departments		80 (7.91)	249.50 (214.00, 266.00)		
	Administrative support department		14 (1.38)	257.00 (211.50, 267.25)		
Multi-department rotation or work experience	No		345 (34.12)	261.00 (217.00, 270.00)	−0.828^b^	0.408
	Yes		666 (65.88)	262.00 (219.00, 270.00)		
Specialized nurse training and certification	No		446 (44.11)	250.50 (216.00, 270.00)	−5.342^b^	<0.001
	Yes		565 (55.89)	266.00 (228.50, 270.00)		
Levels of specialized nurse training	None		446 (44.11)	250.50 (216.00, 270.00)	30.587^a^	<0.001
	Hospital level		276 (27.30)	266.00 (228.25, 270.00)		
	City level		38 (3.77)	260.50 (216.00, 270.00)		
	Provincial level		144 (14.24)	265.50 (225.00, 270.00)		
	National level		107 (10.58)	266.00 (238.00, 270.00)		
Has become an “sharing nurses”	No		491 (48.57)	238.00 (215.00, 269.00)	−9.787^b^	<0.001
	Yes		520 (51.43)	267.00 (244.25, 270.00)		
Duration of “sharing nurses” (years)		1.00 (0.00, 3.00)			0.279^c^	<0.001
Average number of “sharing nurses” services per year		0.00 (0.00, 2.00)			0.271^c^	<0.001
Average monthly income from “sharing nurses” services		0.00 (0.00, 40.00)			0.274^c^	<0.001
Number of night shifts worked at the hospital each week	0		291 (28.78)	265.00 (237.00, 270.00)	8.925^a^	0.258
	1		112 (11.08)	256.50 (220.25, 270.00)		
	2		408 (40.36)	260.00 (216.00, 270.00)		
	3		95 (9.40)	245.00 (216.00, 270.00)		
	4		41 (4.06)	247.00 (216.00, 270.00)		
	5		11 (1.09)	268.00 (227.00, 270.00)		
	6		15 (1.47)	265.00 (216.00, 270.00)		
	7		38 (3.76)	268.00 (216.00, 270.00)		
Nursing in major public health events	No		401 (39.66)	252.00 (216.00, 270.00)	−3.142^b^	0.002
	Yes		610 (60.34)	264.00 (224.00, 270.00)		
Participate in emergency training organized by hospitals and above-level medical institutions and obtain qualifications	No		536 (53.01)	234.00 (215.00, 268.75)	−11.616^b^	<0.001
	Yes		475 (46.99)	268.00 (251.00, 270.00)		
Have undergone nursing skills training for “sharing nurses” organized by hospitals or higher-level medical institutions	No		326 (32.24)	216.00 (199.75, 223.00)	−21.410^b^	<0.001
	Yes		685 (67.76)	268.00 (257.00, 270.00)		
If changes in the patient’s condition are detected, support from the treatment team at the hospital in terms of referral, updating treatment plans, etc.	No		168 (16.62)	213.50 (170.25, 224.75)	−12.897^b^	<0.001
	Yes		843 (83.38)	266.00 (235.00, 270.00)		
Number of nursing research projects and published papers that you have led or participated in		0.00 (0.00, 1.00)			0.106^c^	<0.001
Have a positive intention to become a “sharing nurses”	No		273 (27.00)	215.00 (194.00, 224.00)	−18.181^b^	<0.001
	Yes		738 (73.00)	268.00 (247.75, 270.00)		
Family support	No		121 (11.97)	211.00 (162.50, 233.50)	−11.329^b^	<0.001
	Yes		890 (88.03)	265.00 (228.00, 270.00)		
Support from hospital or department leaders	No		36 (3.56)	182.00 (162.00, 239.25)	−6.251^b^	<0.001
	Yes		975 (96.44)	263.00 (220.00, 270.00)		
Scores for evaluation index system for emergency response competencies of “Internet+” nurses		195.00 (160.00, 200.00)			0.852^c^	<0.001

a, K-independent-sample nonparametric test; b, two independent samples nonparametric test; c, spearman test.

### 3.6 Data collection method

With approval from nursing administrators, the online questionnaire “Questionnaire Star (16)” was distributed by the team members to eligible nurses meeting the inclusion/exclusion criteria at local hospitals. Questionnaires were completed during nurses’ non-clinical work time in a quiet environment. Prior to participation, the study purpose was explained to each nurse. Team members addressed any questions participants had, refraining from providing leading prompts. Completion time was approximately 10 min. All collected data were stored on a password-protected computer accessible only to the research team to prevent unauthorized access or leakage. Following study completion, the data were securely deleted to ensure information confidentiality.

### 3.7 Data entry and analysis

Double checking the data to avoid errors, all information was statistically analyzed by SPSS 26.0. A total of 1,184 questionnaires were collected. Before data analysis, outliers were screened and incomplete responses were excluded. This resulted in 1011 valid questionnaires, yielding an effective response rate of 85.39% (exclusion criteria: serious mismatch between age and job title, *n* = 3; age<18/>65, *n* = 4; age - duration of clinical experience<18/>47, *n* = 25; age - duration of “sharing nurses” < 18/>47, *n* = 52; working years—duration of “sharing nurses” ≤ 4, *n* = 89; total exclusions *n* = 173). Categorical variables within the general characteristics data were summarized as frequencies and percentages; normality of continuous variables was checked before analysis; normally distributed data were presented as mean ± standard deviation and two independent samples t-tests were used for between-group comparisons; non-normally distributed data were presented as median (interquartile range), and nonparametric tests were used for between-group comparisons. Correlations between continuous variables and questionnaire scores were analyzed using the *Pearson* correlation test (where both sets of variables conformed to normal distribution) or *the Spearman* correlation test (where at least one set of variables did not conform to normal distribution); dichotomous variables were analyzed using nonparametric tests; hierarchical and unordered multicategorical data were analyzed using analyses of variance (*ANOVA*), and when the variances were not equal, the nonparametric tests. Univariate analysis was used to analyze the influence of general information on Scores for the core competencies evaluation index system of “sharing nurses,” and the factors that differed in the univariate analysis were included in a multiple linear regression to analyze how general information and emergency response capabilities affected the core competencies of “sharing nurses.” *P*<0.05 was considered statistically significant.

### 3.8 Ethical principles

The study received approval from the Ethics Committee of Anonymous Hospital (approval number: Anonymous), and all hospital administrators and nurses participating in the study gave informed consent. Participants were assured that involvement was voluntary, they could withdraw at any time without penalty, and refusal to participate would not incur any detriment. Prior to data collection, the research team detailed the study purpose to the nurses. All participants were informed that collected data would remain anonymous and confidential.

### 3.9 Validity, reliability, and rigour

Rigorous quality control procedures were implemented throughout this study to minimize potential confounding bias. Both The core competencies evaluation index system of “sharing nurses” and an evaluation index system for emergency response competency of “Internet+” nurses have been previously utilized and validated within Chinese populations, demonstrating good reliability. The researchers rigorously selected the study subjects according to the inclusion and exclusion criteria, and used uniform explanatory language to answer the questions during the information collection period. To prevent duplicate submissions, the online questionnaire platform was configured to allow only one response per device. Upon completion of data collection, responses from the six participating hospitals were compiled and securely stored in an Excel spreadsheet. All data were first independently organized and verified by two researchers and entered into SPSS for statistical analysis to ensure the accuracy, validity and reliability of the data analysis results.

## 4 Results

### 4.1 Samples and characteristics of participants

Overall data from 1,011 nurses from 6 cities were statistically analyzed. Participants had a median age of 36.00 years (34.00, 40.00), overwhelmingly female (*n* = 932, 92.19%), and most commonly had a bachelor’s degree of education (*n* = 960, 94.95%). While 520 participants (51.43%) had become “sharing nurses,” but only 415 (41.05%) have actually provided “sharing nurses” nursing services to patients more than once. Detailed demographic characteristics of participants are presented in Table 1.

### 4.2 Score of the core competencies evaluation index system of “sharing nurses”

The median score of the core competencies evaluation index system of Shandong Province nurses was 261.00 (218.00, 270.00). The Median scores for the individual competency dimensions were as follows:home nursing practice ability: 55.00 (45.00, 55.00);critical thinking ability: 35.00 (28.00, 35.00);communication and coordination ability: 25.00 (20.00, 25.00);management ability: 45.00 (36.00, 45.00); self-protection ability: 50.00 (40.00, 50.00); professional development potential: 56.00 (48.00, 60.00) (Table 2).

**Table 2:** Scores on core competencies and emergency competencies for “sharing nurses.”

Score		Min	Max	Mean	SD	Median (interquartile range)
Scores for the core competencies evaluation index system of “sharing nurses”	Total score	62	270	244.23	32.11	261.00 (218.00, 270.00)
	Home-based nursing practice ability	11	55	50.45	6.55	55.00 (45.00, 55.00)
	Critical thinking ability	11	35	31.94	4.23	35.00 (28.00, 35.00)
	Communication and coordination ability	9	25	22.75	3.08	25.00 (20.00, 25.00)
	Management ability	9	45	40.74	5.61	45.00 (36.00, 45.00)
	Self-protection ability	10	50	45.88	5.99	50.00 (40.00, 50.00)
	Professional development potential	12	60	52.46	8.48	56.00 (48.00, 60.00)
Scores for evaluation index system for emergency response competencies of “Internet+” nurses	Total score	40	200	181.07	24.15	195.00 (160.00, 200.00)
	Preventive competency	16	80	72.45	9.67	78.00 (64.00, 80.00)
	Preparedness competency	6	30	27.39	3.70	30.00 (24.00, 30.00)
	Response competency	14	70	63.29	8.74	69.00 (56.00, 70.00)
	Restorative competency	4	20	17.94	2.67	20.00 (16.00, 20.00)

### 4.3 Score of evaluation index system for emergency response competencies of “Internet+” nurses

The median score of Shandong Province nurses’ “Internet+” nurse emergency response competencies evaluation indicator system was 195.00 (160.00, 200.00). Median scores for the competency dimensions were as follows: preventive competency: 78.00 (64.00, 80.00); preparedness competency: 30.00 (24.00, 30.00); response competency: 69.00 (56.00, 70.00); restorative competency: 20.00 (16.00, 20.00) (Table 2).

### 4.4 Factors influencing the score of the core competencies evaluation index system of “sharing nurses” in Shandong Province

The results of the univariate analysis for each variable are presented in Table 1. Prior to conducting the multiple linear regression analysis, all independent variables were assigned appropriate values (Table 3). The scores of the core competencies evaluation index system of “sharing nurses” were strongly positively correlated with the scores of “Internet+” nurse emergency response competencies evaluation indicator system (*r* = 0.852, *p* < 0.001) (Table 1). The results of multiple linear regression analysis (Table 4) presented that: the higher the level of the hospital, the stronger the core competencies of the nurses (*β* = 0.028, *p* = 0.039); nurses with extroverted personality had better core competencies (*β* = 0.036, *p* = 0.004); nurses who had received the training of nursing competencies of “sharing nurses” were more competent (*β* = 0.203, *p* < 0.001); when providing “sharing nurses” services, nurses who can access to hospital treatment and referral support (*β* = 0.068, *p* < 0.001), support from hospital or department leaders (*β* = 0.037, *p* = 0.004) and family support (*β* = 0.059, *p* < 0.001)scored higher. Meanwhile, the positive willingness of the nurses themselves to practice (*β* = 0.099, *P* < 0.001) as well as emergency response competencies (*β* = 0.628, *P* < 0.001) had a positive effect on their core competencies.

**Table 3:** Assigning values to variables.

Variable	Assigning values
Hospital level	0 = Second-class A; 1 = Third-class B; 2 = Third-class A
Age	Import original value
Personality	0 = Introverted; 1 = Extroverted
Title	0 = Junior Nurse; 1 = Senior Nurse; 2 = Charge Nurse; 3 = Associate Nurse Practitioner; 4 = Chief Nurse
Position	0 = None; 1 = Deputy head nurse; 2 = Head nurse; 3 = Head nurse of major department; 4 = Deputy director of nursing department; 5 = Director of nursing department
Duration of clinical experience (years)	Import original value
Work department	(0,0,0,0,0,0) = Internal medicine; (0,1,0,0,0,0) = Surgery medicine; (0,0,1,0,0,0) = Gynecology and pediatrics; (0,0,0,1,0,0) = ICU and emergency department; (0,0,0,0,1,0) = Outpatient clinics, medical technology, and auxiliary departments; (0,0,0,0,0,1) = Administrative support department
Levels of specialized nurse training	0 = None; 1 = Hospital level; 2 = City level; 3 = Provincial level; 4 = National level
Duration of “sharing nurses” (years)	Import original value
Average number of “sharing nurses” services per year	Import original value
Average monthly income from “sharing nurses” services	Import original value
Nursing in major public health events	0 = No; 1 = Yes
Have undergone nursing skills training for “sharing nurses” organized by hospitals or higher-level medical institutions	0 = No; 1 = Yes
If changes in the patient’s condition are detected, support from the treatment team at the hospital in terms of referral, updating treatment plans, etc.	0 = No; 1 = Yes
Number of nursing research projects and published papers that you have led or participated in	Import original value
Have a positive intention to become an “sharing nurses”	0 = No; 1 = Yes
Family support	0 = No; 1 = Yes
Support from hospital or department leaders	0 = No; 1 = Yes
Scores for evaluation index system for emergency response competencies of “Internet+” nurses	Import original value

**Table 4:** Multiple regression analysis of the factors influencing the core competencies of “sharing nurses.”

Variable	*B*	*SE*	*β*	*t*	*P*
Constant	56.411	6.647	—	8.487	<0.001
Hospital level	1.074	0.518	0.028	2.071	0.039
Age	0.139	0.209	0.019	0.666	0.506
Personality	2.350	0.805	0.036	2.918	0.004
Title	−0.198	0.763	−0.004	−0.260	0.795
Position	−0.426	0.738	−0.008	−0.577	0.564
Duration of clinical experience	0.010	0.197	−0.001	−0.050	0.960
Levels of specialized nurse training	−0.484	0.297	−0.021	−1.626	0.104
Duration of “sharing nurses”	−0.183	0.293	−0.009	−0.626	0.531
Average number of “sharing nurses” services per year	0.043	0.178	0.009	0.243	0.808
Average monthly income from “sharing nurses” services	0.004	0.013	0.013	0.343	0.732
Nursing in major public health events	−1.282	0.843	−0.020	−1.521	0.129
Participate in emergency training organized by hospitals and above-level medical institutions and obtain qualifications	0.810	0.885	0.013	0.916	0.360
Have undergone nursing skills training for “sharing nurses” organized by hospitals or higher-level medical institutions	13.922	1.152	0.203	12.086	<0.001
If changes in the patient’s condition are detected, support from the treatment team at the hospital in terms of referral, updating treatment plans, etc.	5.891	1.231	0.068	4.784	<0.001
Number of nursing research projects and published papers that you have led or participated in	0.117	0.289	0.006	0.405	0.685
Have a positive intention to become a “sharing nurses”	7.171	1.150	0.099	6.238	<0.001
Family support	5.869	1.391	0.059	4.219	<0.001
Support from hospital or department leaders	6.417	2.246	0.037	2.857	0.004
Scores for evaluation index system for emergency response competencies of “Internet+” nurses	0.835	0.024	0.628	34.645	<0.001
Work department: internal medicine	0				
Surgery medicine	−0.873	0.970	−0.012	−0.901	0.368
Gynecology and pediatrics	1.397	1.395	0.013	1.001	0.317
ICU and emergency department	−0.905	1.381	−0.009	−0.655	0.512
Outpatient clinics, medical technology and auxiliary departments	−0.641	1.543	−0.005	−0.415	0.678
Administrative support department	−2.065	3.459	−0.008	−0.597	0.551

## 5 Discussion

The core competencies of nursing is defined as “the ability to meet the needs of individuals requiring care through logical thinking and precise nursing skills.” The structure of nursing competency encompasses four abilities: understanding needs, providing care, collaborative practice, and supporting decision-making. Which are closely interrelated and utilized in all types of nursing practice settings (17). The American Organization of Nurse Leaders identified key areas for developing core competencies and fostering self-development, including: the ability to establish and maintain relationships, communication and coordination skills, leadership and management competencies, understanding of the healthcare environment, professionalism, and proficient nursing skills (18). Managerial competencies are context-sensitive, influenced by the complexities of departments, teams and organizations. Effective managerial competence forms the foundation for successful and efficient healthcare organizations. It not only enhances the quality of care but also contributes to higher levels of nurse dedication, reduced turnover rates, and the cultivation of positive nursing attitudes (19). The development of the core competencies evaluation index system of “sharing nurses” provides healthcare organizations and nursing managers with assessment tools to accurately understand, describe, analyze and evaluate the competencies level of nurses. This system is crucial for improving the service quality of China’s “sharing nurses,” and offers a “Chinese model” for nurses globally seeking to broaden their career paths. By systematically assessing and analyzing the influencing factors on the comprehensive nursing competencies of “sharing nurses,” coupled with standardized training. We can not only elevate their professional level and the quality of their nursing services, but also guarantee the safety of patients and enhance their care experience. This approach fosters progress across the entire nursing profession towards greater professionalism and efficiency.

### 5.1 Chinese “sharing nurses” demonstrate high levels of core competencies

The core competencies of Chinese nurses are assessed at a high level. In this study, nurses demonstrated strong proficiency in home nursing practice skill, critical thinking skill, communication and coordination skill, management skill, self-protection ability, while also exhibiting considerable potential for professional development. This finding suggests that following government administrative intervention, medical institutions broadened the scope and location of nurses’ practice in a very short period of time, and actively carried out the pilot work of “sharing nurses” nursing service to promote nurses’ provision of home-based nursing service to patients, and the core competencies of “sharing nurses” were rapidly improved. This mode of practice, which was previously restricted or ambiguous, demonstrates the pivotal role of government policy in advancing the nursing profession (20). The construction and application of core competencies evaluation index systems across various nursing specialties have received widespread attention (21), which not only provide an effective means to assess nurses’ professionalism but also establish a foundation for expanding their career paths. Cultivating core competencies play a vital role in nurses’ career development. These competencies not only influence their career development, but also directly impact clinical performance. Studies have shown that there is a significant positive correlation between nurses’ core competencies and their career success. Enhancing core competencies can improve nurses’ job satisfaction and professional competitiveness (22). The development of core competencies was an ongoing process throughout a nurse’s career, and nurses’ core competencies, especially critical thinking skills, positively influence their impact within nursing practice (23). Nurses are faced with the challenge of enhancing their professional competencies as the practice environment undergoes rapid change, and continuing nursing education and skills training are key, however, nurses are constrained by time, resources and costs when engaging in their own development (24, 25). Therefore, the development of a scientific continuing education and training program, especially for the core competencies of “sharing nurses,” can effectively enhance their practice confidence.

### 5.2 Chinese “sharing nurses” demonstrate high levels of “Internet+” emergency response abilities

Chinese nurses’ “Internet+” emergency response abilities are at a high level. In this study, the nurses’ preventive, preparatory, reactive, and restorative competence were strong, which may be related to the higher titles (76.56% of intermediate and higher titles) and substantial work experience of the nurses collected in this study. Nurses are often alone when they perform “sharing nurses” services in patients’ homes, and this practice model necessitates that nurses possess not only core professional competencies but also the ability to deal with emergencies, such as sudden changes in patient’s condition (26). Enhancing nurses’ emergency response abilities not only strengthens their core competencies, but also expands their professional roles and spaces. Furthermore, by continually elevating the professional status and influence of nursing, nurses can play a more critical role in public health crises (27). A study focusing on emergency response to infectious diseases revealed that although the emergency response capacity of Chinese nurses was in the middle to high level, nearly one-fifth of nurses still lacked sufficient rehearsal and training experience (28), which suggests that despite the “Internet+” technology can provide more learning opportunities for nurses, multi-scenario rehearsal and training are still the key factors to improve emergency response capacity. In 2023, three years after the policy was approved, the emergency response capacity of Chinese “sharing nurses” is still deficient in terms of prevention and recovery. Nurses who are married and have experience in home-based care services and emergency response training show stronger emergency response capacity (29). Additionally, nurses who have participated in frequent emergency response training perform more effectively in emergency response (30), suggesting that regular emergency response training and drills are needed in the future to effectively improve their emergency response ability and resuscitation confidence. Therefore, it is recommended that medical institutions optimize their training programs to further enhance nurses’ emergency response abilities by setting up simulation scenarios. In conclusion, “sharing nurses” can continuously develop their emergency response ability by participating in frequent and high-quality trainings throughout their career, so as to ensure that they are capable of responding to various emergencies to ensure patient safety when they are performing nursing operations at patients’ homes.

### 5.3 A social-ecosystems perspective on the influencing factors of core competencies among Chinese “sharing nurses”

The core competencies of “sharing nurses” are influenced by a variety of factors, including hospital level, the support of the hospital leadership and the support of the team in treatment at the macro level, the support of the family at the meso level, and the personality, the subjective willingness, the training experience, and the ability to respond to emergencies at the micro level.

#### 5.3.1 The macro and meso level

At the macro level, a novel finding emerged: the core competencies of “sharing nurses” were lower in Level Second-class A hospitals than in Level Third-class B, and stronger in Level Third-class B hospitals than in Level Third-class A hospitals, which suggesting that there is a correlation between the core competencies of “sharing nurses” and the level of the hospitals in which they work. Typically, higher-tier hospitals possess greater technical advantages in resource allocation, stronger multidisciplinary collaboration and resource integration capabilities, and provide nurses with ongoing exposure to cutting-edge skills, thereby fostering stronger core competencies (31). However, the medical nature of different levels of hospitals is different, Second-class A hospitals often admit and treat patients with acute and critical illnesses, and nurses have a larger workload, compared with nurses in Third-class B hospitals, they do not have more energy to become a “sharing nurses” (32). Policy support from hospitals and departments is crucial for enhancing core competencies, and studies indicates that performance bonuses, work schedules, professional development resources and leader attitudes influence nursing practitioners’ attitudes, knowledge and skills in their specialty areas (33, 34). It suggests that in the future, healthcare organizations should implement incentive policies and strengthen treatment team support to increase the motivation of “sharing nurses” to practice, and ensure that they can receive hospitals’ support regarding referrals and updating of treatment protocols if they notice a change in the patient’s condition while practicing in the community and at home. At the meso level, nurses with greater family support demonstrate stronger core competencies. Nurses are predominantly female, and when there is a conflict between work and family, they often face both physical and psychological pressure. If family members are able to provide nurses with continuous emotional support and share some of the housework and childcare responsibilities, nurses’ burnout can be alleviated (35). In conclusion, “work-family” support can not only mitigate the negative impact of nurses’ high workload, but also improve their work engagement, thus enhancing their core competencies and achieving career success.

#### 5.3.2 The micro level

At the micro level, intergroup comparisons revealed that nurses who had become “sharing nurses” had stronger core competencies, suggesting that practical experience is the basis for improving core competencies in nursing specialties (36). Through continuous accumulation of nursing experience and self-reflective learning in complex cases, nurses can establish significant advantages in core competencies such as technical operation and emergency decision-making. Extraverted personality nurses have stronger core competencies than introverted nurses, the reason may be that extraverted personality nurses have higher levels of occupational well-being and psychological resilience, the psychological workload such as emotional stress is lower than that of introverted personality nurses, and they can maintain positive emotions more effectively under work pressure to provide patients with better nursing care, and therefore have stronger core competencies (37). The higher the subjective willingness of nurses, the higher the level of their core competencies, which may be attributed to the fact that nurses who are interested in carrying out “sharing nurses” services are more willing to learn relevant knowledge and receive skills training to meet the competency demands of their service delivery (38). Providing training positively impacts nurses’ professional performance. Trained nurses show improvements in knowledge, operational proficiency, and preventive capabilities, thereby enhancing their competencies to ensure patient safety and reduce complication rates (39). Since nursing services are provided in the patient’s home, skill training of nurses and standardization of service processes are crucial prerequisites to ensure the quality of care and patient safety (40). Consequently, providing training to nurses to help them rapidly master new technologies is essential for improving their core competencies (41). This study shows that the stronger the emergency response ability of “sharing nurses,” the stronger the core competencies. As an integral component of core competencies, nurses’ emergency response capacity plays a vital role in effectively managing acute and critical patients, safeguarding patient safety, improving medical rescue quality, and optimizing public health emergency management systems (42), which suggests that healthcare institutions need to cultivate the emergency response abilities as one of the important core competencies when formulating the training program. Collectively, these findings can help medical institutions analyze the core competencies level of “sharing nurses” based on the influencing factors, construct education and training programs in a targeted manner, and further select appropriate educational paths according to the individual characteristics of nurses, in order to provide better nursing services to home-based patients, and provide reference for nurses to broaden their career paths.

## 6 Limitations

First, the cross-sectional design used in this study was not able to explain the causal relationships between the variables, and it is recommended that future researchers use a longitudinal design to explore the trajectories of the variables. Second, this study included subjectively reported data from nurses, and despite quality control measures were implemented, relying on self-reported data may introduce subjectivity bias, which may produce some degree of recall bias and social desirability bias, and future studies should incorporate data from face-to-face qualitative interviews in order to improve the authenticity and reliability of the findings. Third, Shandong Province as one of the earliest provinces in China to be allowed by the government to carry out the pilot program of “sharing nurses” service, has carried out the training earlier than other provinces, and the nursing procedures are relatively mature, so the proportion of “sharing nurses” in the sample of nurses collected and the scores on the questionnaire are higher. However, these data may not represent the overall competencies level of nurses across China, as many provinces or cities had not yet been allowed to provide this service at the time of publication. Hence, future research could explore the applicability of our findings in the context of different national healthcare systems, while also examining how “sharing nurses” services might address potential legal, regulatory, and ethical challenges they may encounter.

## 7 Conclusion

Although Chinese “sharing nurses” demonstrate relatively high levels of core competencies and emergency response capabilities, but only 79.81% have actually delivered these services. The rate of nurses receiving specialized training in the core competencies and emergency response capabilities required is not high. While “sharing nurses” services have positively impacted healthcare efficiency, home-based patients still require time to gradually accept this novel and convenient care model. In the future, healthcare institutions should actively provide standardized training to nurses aligned with national policies, increase nurses’ willingness to become “sharing nurses.” Additionally, encourage them to deliver these services and safeguard the quality of life and safety of home-based patients with the support of hospital’s treatment teams. Additionally, the government and healthcare institutions should intensify publicity and support for “sharing nurses” services, expand medical insurance coverage scope and increase reimbursement rates to reduce the financial burden of home-based patients and accelerate widespread adoption of this service model.

## Data Availability

The original contributions presented in the study are included in the article/supplementary material, further inquiries can be directed to the corresponding author/s.
